# Hydrothermal Synthesis, Structural Characterization, and Interaction Mechanism with DNA of Copper(II) Complex Containing 2,2′-Bipyridine

**DOI:** 10.1155/2018/8459638

**Published:** 2018-05-22

**Authors:** Ting Liu, Yi-An Wang, Qing Zang, Guo-Qing Zhong

**Affiliations:** School of Material Science and Engineering, Southwest University of Science and Technology, Mianyang 621010, China

## Abstract

A Cu(II) complex [Cu(bipy)(H_2_O)_2_(SO_4_)]_*n*_ (bipy = 2,2′-bipyridine) was synthesized by hydrothermal method and characterized structurally by elemental analyses, single crystal X-ray diffraction, infrared spectra, and thermogravimetry and differential scanning calorimetry. The Cu(II) was hexacoordinated by two N atoms from bipy, two O atoms from different sulfate radical anions, and two O atoms from two water molecules, forming a slightly distorted octahedral geometry, and bridged by sulfato groups into polymeric chains. Under the condition of physiological pH, the interaction mechanism between the complex and hsDNA was explored with acridine orange as a fluorescence probe by spectroscopic methods. The binding modes between the complex and hsDNA were the electrostatic and embedded modes.

## 1. Introduction

Design and synthesis of organometallic complexes have become an active research area because of their novel topologies, fascinating functionalities, special properties, and potential applications, such as biomedical utilization, multifunctional materials, molecular adsorption, gas storage, catalysis, magnetism, and so on [1–6]. Chemists have synthesized many organic-inorganic hybrid materials with nitrogen heterocyclic compounds as organic building blocks [7–10]. Trace element copper plays an important role in endogenous oxidative DNA damage associated with aging and cancer [11, 12]. Cu(II) complexes have many bioactivities such as antitumor [13, 14], antimicrobial [15–17], and oxidation of ascorbic acid in the presence of oxygen [18]. In addition, Cu(II) complexes can bind to DNA through noncovalent and covalent interactions [19]. Many researchers found that Cu(II) complexes had potential in the treatment of cancers and other diseases [20]. 2,2′-Bipyridine is a potential antitumor agent and often acts as ancillary ligand to strengthen the binding ability of a complex through enhancing the molecule planarity [21]. The complexes of 2,2′-bipyridine and its derivatives have been reported by a number of authors [22, 23]. The method of hydrothermal synthesis has been used to produce various solids, such as oxide ceramics, microporous crystals, metal complexes, nanomaterials, and so forth [24–29]. In particular, the molecular structures obtained by this method are unexpected compared with those obtained by the common solution method [30].

The modes of noncovalent interaction for metal complexes with DNA include intercalation, electrostatic effect, groove binding, and so on, and the effectiveness mainly depends on the binding modes and affinities between complexes and DNA [31–34]. There is continuing interest in some metal complexes that interact with DNA [35]. Furthermore, the studies of interaction of metal coordination polymers with DNA have been of great interest [36, 37]. However, examples of such metal coordination polymers are still few. Therefore, it is of great significance to explore the binding modes of DNA with metal coordination polymers containing rigid ligands.

We herein report the X-ray single crystal structure, Fourier transform infrared spectra (FTIR), and thermogravimetry and differential scanning calorimetry (TG-DSC) of the Cu(II) complex containing 2,2′-bipyridine, which is synthesized by hydrothermal method, and explore its bioactivities with herring sperm DNA (hsDNA) using acridine orange (AO) as a fluorescence probe by spectral methods.

## 2. Materials and Methods

### 2.1. Materials

All chemicals were of analytical reagents and used as received without further purification. 2,2′-Bipyridine and AO were purchased from Alfa Aesar, and copper sulfate pentahydrate and other reagents were purchased from Merck. The hsDNA was purchased from Sigma Biological Co., its purity was monitored by the ratio of absorbance at 260–280 nm, and the ratio of 1.8–1.9 indicated that the hsDNA was free from protein. The hsDNA was dissolved in double-distilled water with 50 mmol·L^−1^ sodium chloride and dialyzed at 4°C for 48 h [38]. The hsDNA concentration was measured by UV-Vis at 260 nm. Tris-HCl buffer solution (pH 7.40) was prepared by using triple-distilled water.

### 2.2. Physical Measurements

The C, H, and N in the complex were analyzed with a Vario EL CUBE elemental analyzer, and the copper was determined by EDTA titration. FTIR spectra were obtained with KBr pellets on a Perkin-Elmer Spectrum One-Spectrometer in the range 4000–400 cm^−1^. The thermal analysis was performed by a SDT Q600 thermogravimetric analyzer from 30 to 800°C at a heating rate of 10°C·min^−1^ under air flow of 100 mL·min^−1^. UV-Vis spectra in Tris-HCl buffer solution (pH 7.40) were measured with a Unico spectrophotometer (UV-2102) in the range 200–600 nm. Fluorescence spectra were recorded on a PE LS-55 spectrofluorophotometer. Viscosities were measured with an Ubbelohde capillary viscometer having diameters of 0.40–0.50 nm and 0.50–0.60 nm, respectively. The viscometers were selected on the basis of the flow time of the complex, and the flow time was at least 120 s.

### 2.3. Synthesis of the Title Complex

2,2′-Bipyridine (0.2 mmol, 31.7 mg) and NaOH (0.2 mmol, 8.0 mg) were dissolved in a minimum amount of distilled water. Copper sulfate (0.2 mmol, 51.3 mg) was added to the above solution, and the volume of the mixed solution was increased to 18 mL with distilled water. The mixture was transferred to a 30 mL Teflon-lined stainless steel reactor and heated to 140°C for 72 h, and then, it was cooled at a rate of 10°C·h^−1^ to room temperature. Blue stick single crystals suitable for X-ray diffraction analysis were obtained. The crystals were washed by a small amount of distilled water and anhydrous ethanol and dried naturally (yield 84%). Anal. Calc. (%) for CuC_10_H_12_N_2_O_6_S: C, 34.14; H, 3.44; N, 7.96; Cu, 18.06. Found (%): C, 34.12; H, 3.30; N, 7.77; Cu 18.14.

### 2.4. X-Ray Crystallography

A blue crystal with dimensions 0.374 mm × 0.199 mm × 0.117 mm was installed on a Bruker Apex II CCD diffractometer with graphite monochromated Mo K_*α*_ radiation (*λ* = 0.71073 Å). Diffraction data were collected at 296(2) K in the *θ* range 3.273–27.639°. The programs of the SHELXL-97 and SHELXTL-97 were used for the structure determination and refinement [39, 40]. The structure was solved by direct methods, and all nonhydrogen atoms were obtained from the difference Fourier map and subjected to anisotropic refinement by full-matrix least squares on *F*^2^. Crystallographic data have been deposited with the Cambridge Crystallographic Data Centre, CCDC, UK. Copies of the data can be obtained free of charge on quoting the depository CCDC-1028718 for the title complex (deposit@ccdc.cam.ac.uk, http://www.ccdc.cam.ac.uk).

### 2.5. Procedures of Biological Activity

The experimental methods of biological activity were mainly referred to [38, 41]. The specimens for absorption and fluorescence were obtained through diluting the stock solutions of the title complex (abbreviated as Cu-bipy) and hsDNA with Tris-HCl buffer solution to the required concentrations. Under the condition of the fixed Cu-bipy concentration and changing only the hsDNA concentration, the spectra of UV-Vis and fluorescence were tested with the quartz cuvettes of 1 cm. The excitation wavelength of the fluorescence measurement was 411.7 nm.

The samples of viscosity measurement were filled into the cleaned and dried viscometers. A thermostat was used to keep the temperature constant with the deviations within ±0.01°C. Double-distilled water was used in the calibration experiments, and the viscosity of pure water was derived from Lange's Handbook of Chemistry [42]. The time was recorded on a digital stopwatch with the deviations within ±0.01 s, and the average deviation of the three experimental results was within ±0.2 s. The hsDNA of 1.0 × 10^−5^ mol·L^−1^ was mixed with different concentrations of Cu-bipy. The flow time was recorded at 20 ± 0.1°C after the reaction mixture was placed in the darkness for 0.5 h. The relative viscosities of hsDNA were measured with molar ratio (*r* = *c*(Cu-bipy)/*c*(hsDNA)) from 0.0 to 4.0 at atmospheric pressure and 15°C ambient temperature.

## 3. Results and Discussion

### 3.1. Crystal Structure Analysis

**Figure 1:**
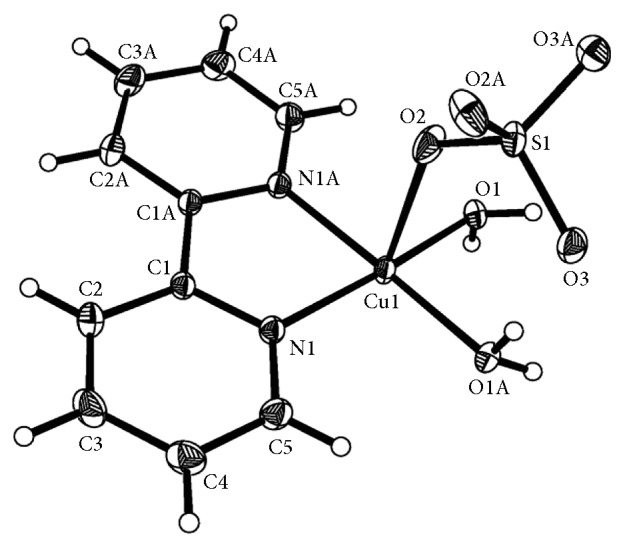
Molecular structure of the title complex.

The molecular structure diagram of the complex is shown in Figure 1. The crystallographic data and structure refinement parameters are given in Table 1, and the selected bond distances and angles are shown in Table 2.

**Figure 2:**
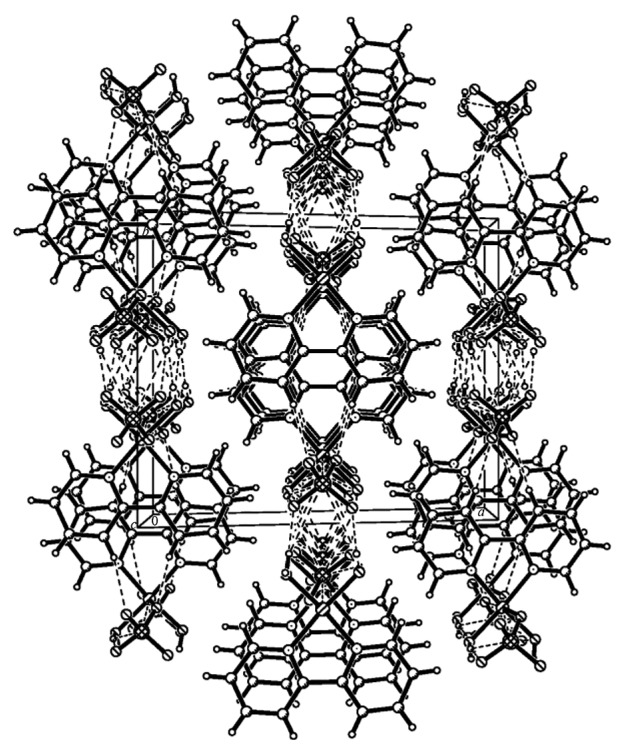
Crystal packing diagram of the title complex.

**Figure 3:**
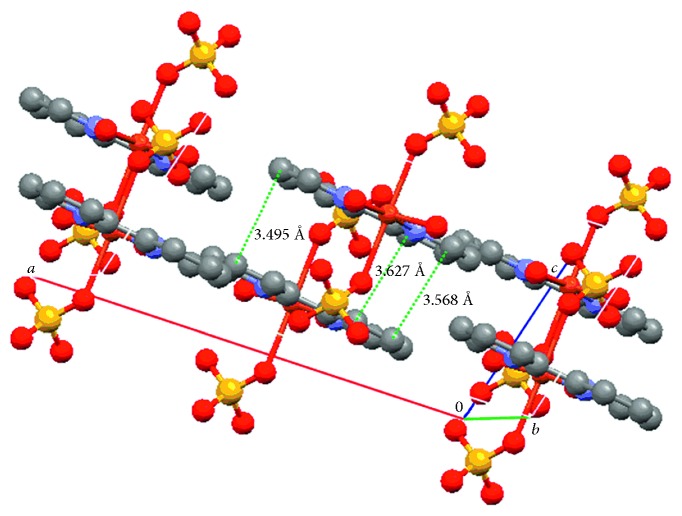
Weak *π*–*π* stacking interactions of the title complex.

The unit of the complex is composed of one Cu(II), one 2,2′-bipyridine, two water molecules, and one sulfate radical anion. The Cu(II) is hexacoordinated by two O atoms from the coordinated water molecules and two pyridyl N atoms from bipy which are located at equatorial sites and two O atoms from different bidentate bridging sulfato groups which are located at axial positions. The coordination configuration is a slightly distorted octahedron, and the Cu(II) is bridged by sulfato groups into polymeric chains. The distances of Cu–O with the aqua ligands and the sulfato group are 1.9728 and 2.455 Å, respectively, and the distances of Cu–N are 1.9947 Å. Because of Jahn–Teller effect of Cu(II) with *d*^9^ electron configuration, the axial distances of Cu(1)–O(2) and Cu(1)–O(2)^#1^ are stretched. As shown in Figure 2, the generated chains extend along (001) plane direction, and the crossing of the chelate bipy forms the polymeric chains formulated as [Cu(bipy)(H_2_O)_2_(SO_4_)]_*n*_. In Figure 3, the molecular structure shows the existence of face-to-face *π*–*π* stacking weak interaction. The interplanar distances of 3.495–3.627 Å between two adjacent mirror planes of bipy are normal for weak *π*–*π* interaction. The hydrogen bond lengths and bond angles are given in Table 3, and the molecules of the complex are linked together by intermolecular hydrogen bonds. It is obvious that the formation of the interchain hydrogen bonds is related to the layers parallel to the (100) plane. A weak hydrogen bond with *d*(C⋯O) = 3.282 Å is formed between the outer C–H bonds of one chain and the coordinated sulfato O atoms of the adjacent chain, and the chains are stabilized by interchain *π*–*π* interaction and interchain C–H⋯O hydrogen bonds [23]. Each water molecule in the complex nearly forms a linear intrachain and interchain hydrogen bonds with the uncoordinated sulfato O atoms.

### 3.2. FTIR Spectroscopy

**Figure 4:**
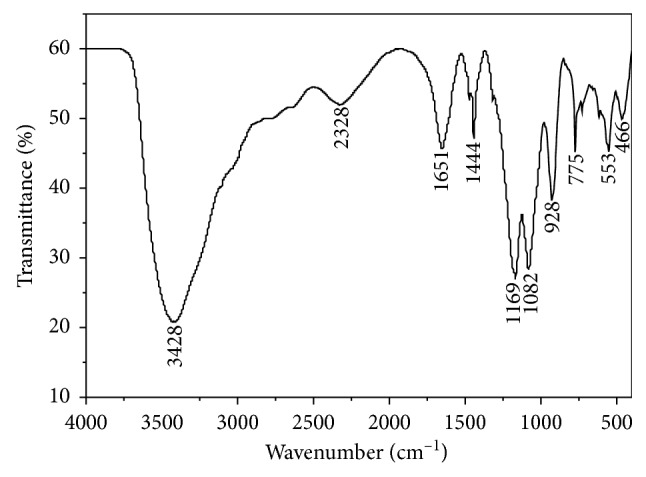
FTIR spectrum of the title complex.

The FTIR spectrum of the complex is shown in Figure 4. Few number of the absorption bands in the FTIR spectrum means that the symmetry of the complex is very good. A wide intense absorption band around 3428 cm^−1^ is due to stretching vibration of hydroxyl [43, 44]. This indicates that there are the coordinated water or lattice water molecules in the complex. The band corresponding to the stretching vibration of the cumulative double bond (C=C–C=C) of pyridine ring is situated at 2328 cm^−1^. The absorption peaks at 1651 and 1444 cm^−1^ are assigned to the stretching vibrations of the C=N and C=C bonds, respectively [45].

As a free anion, sulfate has tetrahedral symmetry, whereas, if sulfate forms a bidentate binuclear (bridging) complex, the symmetry is lowered and the band splits into two bands [46]. As shown in Figure 4, the FTIR spectrum of the complex makes out peaks at 1169 and 1082 cm^−1^. The absorption peaks around 928 and 775 cm^−1^ are assigned to the rocking and wagging vibrations of the hydroxyl, which indicate the existence of the coordinated water molecules in the complex [47]. As a result of the formation of the Cu–N bond, the corresponding C–N bond becomes so weak that disappeared in the FTIR spectrum [48]. The absorption peaks at 553 and 466 cm^−1^ are assigned to the Cu–N bond and Cu–O bond, respectively [49], which agrees with the X-ray crystal structure of the complex.

### 3.3. Thermal Analysis

**Figure 5:**
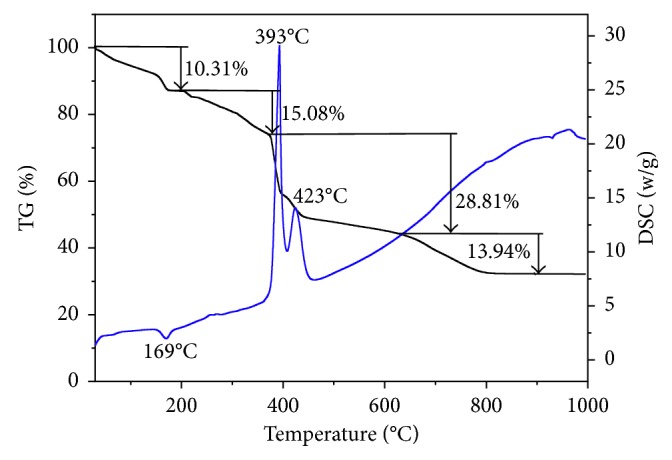
TG-DSC curves of the title complex.

The TG-DSC curves of the title complex are shown in Figure 5, and there are one endothermic peak and two exothermic peaks in the DSC curve. The endothermic peak at 169°C is accompanied by obvious mass loss, and the sample loses two H_2_O molecules. The experimental mass loss (10.31%) is close to the calculated one (10.24%). Due to the high temperature of water loss, the molecules should be the coordinated water. After the water molecules are lost, the complex becomes [Cu(C_10_H_8_N_2_)(SO_4_)]. The sequential exothermic peaks at 393 and 423°C in the DSC curve correspond to the oxidation and decomposition of bipy, the decomposition product of this step is CuSO_4_, and the mass loss of 43.89% is in agreement with the calculated result of 44.39%. The mass loss remains constant until ca. 800°C, the final remnant mass is 31.86%, and the residue is Cu_2_SO_4_ (calculated as 31.72%).

### 3.4. Biological Activity

#### 3.4.1. Binding Ratio

**Figure 6:**
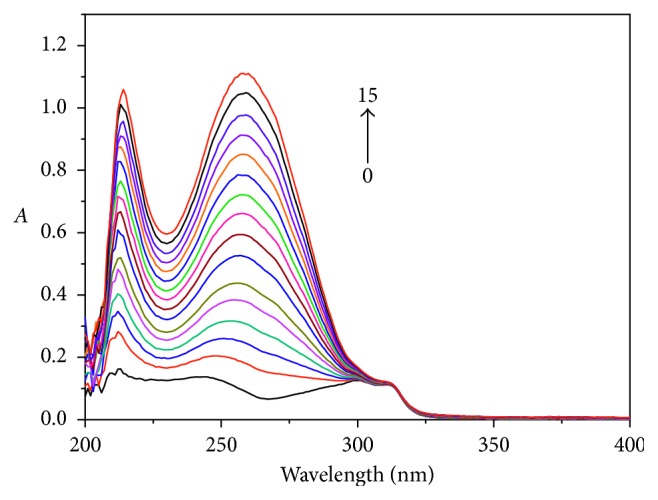
Influence of hsDNA on the UV-Vis spectra of Cu-bipy (pH 7.40). From curves 0–15, *c*(Cu-bipy) = 4.0 × 10^−6^ mol·L^−1^; *c*(hsDNA) = 0.00, 0.17, 0.33, 0.50, 0.67, 0.83, 1.00, 1.17, 1.33, 1.50, 1.67, 1.83, 2.00, 2.17, 2.33, and 2.50 × 10^−6^ mol·L^−1^, respectively.

**Figure 7:**
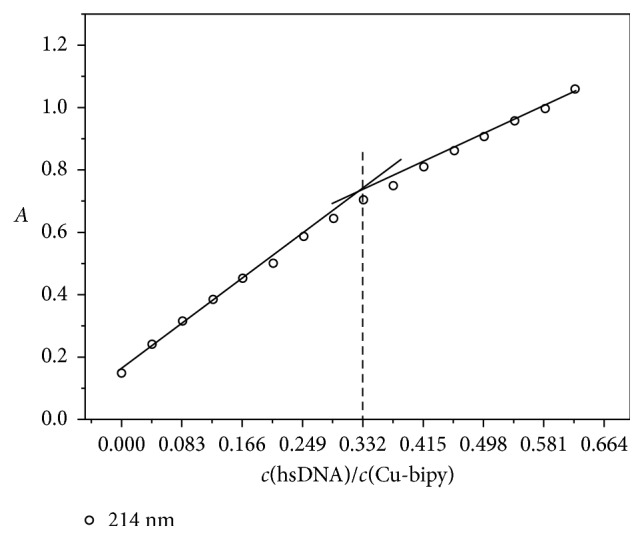
Mole ratio plots of Cu-bipy with hsDNA (pH = 7.40, *λ* = 214 nm), *c*(Cu-bipy) = 4.0 × 10^−6^ mol·L^−1^.

As shown in Figure 6, the UV-Vis spectra were obtained by determination of the Cu-bipy solution with an independent variable of hsDNA concentration. The wavelength which is obtained from Figure 6 and used in the mole ratio method is 214 nm, and the binding ratio [*n*(Cu-bipy) : *n*(hsDNA) = 3 : 1] is shown in Figure 7.

#### 3.4.2. Double Reciprocal Method

The double reciprocal equation [50] is listed as follows to express the relationship between Cu-bipy and hsDNA:

(1) $$\begin{array}{c} {\left(A-A_{0}\right)}^{-1}=A_{0}^{-1}+{\left[K^{⊝}\cdot A_{0}\cdot c\left(\mathrm{hsDNA}\right)\right]}^{-1}. \end{array}$$

**Figure 8:**
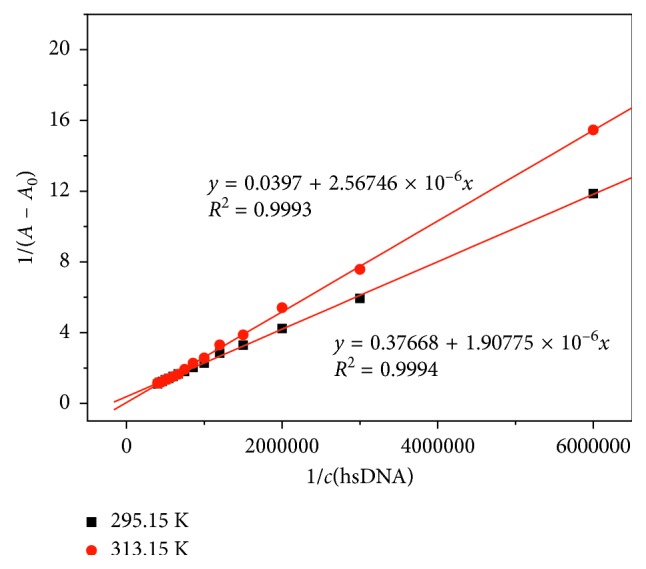
Double reciprocal plots of hsDNA-Cu-bipy at 295.15 K and 313.15 K (pH 7.40). *c*(Cu-bipy) = 4.0 × 10^−6^ mol·L^−1^; *c*(hsDNA) = 0.00, 0.60, 1.20, 1.80, 2.40, 3.00, 3.60, 4.20, 4.80, 5.40, 6.00, 6.60, 7.20, 7.80, and 8.40 × 10^−6^ mol·L^−1^, respectively.

In (1), *c*(hsDNA) is the hsDNA concentration, *A* and *A*_0_ are the absorbance of Cu-bipy in the presence and lack of hsDNA, respectively, and *K*^⊝^ is the binding constant of hsDNA-Cu-bipy. In Figure 8, 1/*c*(hsDNA) is used as an abscissa and 1/(*A* − *A*_0_) is used as an ordinate. The binding constants are, respectively, calculated: *K*^⊝^ (295.15 K) = 1.97 × 10^5^ L·mol^−1^ and *K*^⊝^ (313.15 K) = 1.55 × 10^4^ L·mol^−1^. As is known to all, there are some ways in which macromolecule bind to small molecule, including hydrogen bond, van der Waals force, hydrophobic force, electrostatic interaction, and so on. According to the following equations, we can calculate a series of thermodynamic parameters (Δ_r_*H*_m_^⊝^, Δ_r_*S*_m_^⊝^, and Δ_r_*G*_m_^⊝^) to confirm the interaction forces.

(2) $$\begin{array}{c} \begin{array}{l} \ln\frac{K_{2}^{⊝}}{K_{1}^{⊝}}=\frac{\Delta_{r}H_{m}^{⊝}\left(\left(1/T_{1}\right)-\left(1/T_{2}\right)\right)}{R},\\ \Delta_{r}G_{m}^{⊝}=-RT\;\;\ln\;\;K^{⊝},\\ \Delta_{r}G_{m}^{⊝}=\Delta_{r}H_{m}^{⊝}-T\Delta_{r}S_{m}^{⊝}, \end{array} \end{array}$$

where *T*_1_ is 295.15 K, *T*_2_ is 313.15 K, and Δ_r_*H*_m_^⊝^ and Δ_r_*G*_m_^⊝^ are the standard molar reaction enthalpy and the standard molar reaction Gibbs free energy, respectively. The calculated results in Table 4 indicate that the interaction between Cu-bipy and hsDNA is driven by entropy [51]. The values of Δ_r_*H*_m_^⊝^ and Δ_r_*G*_m_^⊝^ indicate that this is an exothermic reaction, and there is a spontaneous interaction between Cu-bipy and hsDNA.

#### 3.4.3. Competitive Binding Experiments

**Figure 9:**
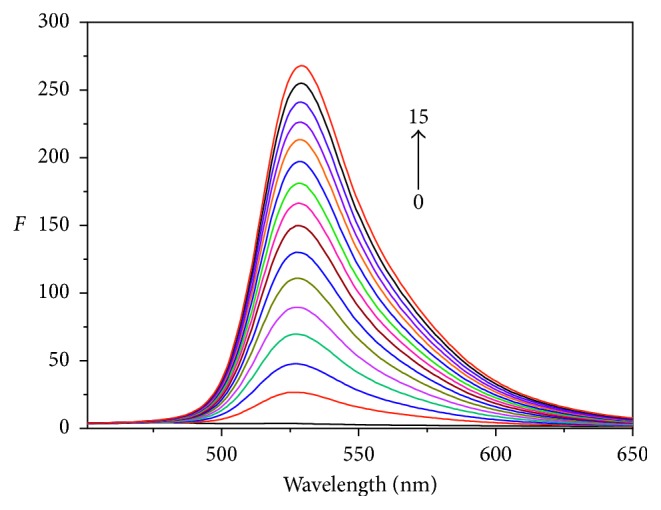
Emission spectra of hsDNA-Cu-bipy mixture in different concentrations of AO (pH = 7.40, *λ*_ex_ = 411.7 nm). From curves 0–15, *c*(hsDNA-Cu-bipy) = 1.0 × 10^−5^ mol·L^−1^; *c*(Cu-bipy) = 0.00, 0.33, 0.67, 1.00, 1.33, 1.67, 2.00, 2.33, 2.67, 3.00, 3.33, 3.67, 4.00, 4.33, 4.67, and 5.00 × 10^−6^ mol·L^−1^, respectively.

**Figure 10:**
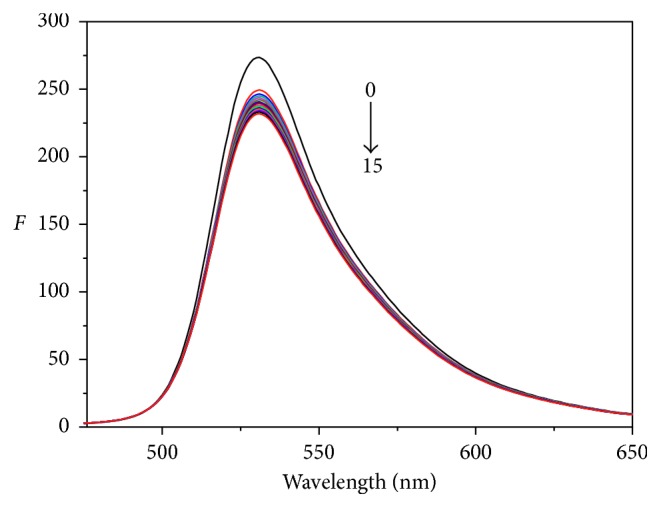
Emission spectra of hsDNA-AO mixture in different concentrations of Cu-bipy (pH = 7.40, *λ*_ex_ = 411.7 nm). From curves 0–15, *c*(hsDNA-AO) = 1.0 × 10^−5^ mol·L^−1^; *c*(Cu-bipy) = 0.00, 0.33, 0.67, 1.00, 1.33, 1.67, 2.00, 2.33, 2.67, 3.00, 3.33, 3.67, 4.00, 4.33, 4.67, and 5.00 × 10^−6^ mol·L^−1^, respectively.

AO as a fluorescence probe was widely used to study the binding way between small molecule and DNA [38], and it can embed between two adjacent base pairs of DNA helix and enhance the fluorescence intensity. As the concentration of AO (Cu-bipy) increases, the fluorescence intensity of Cu-bipy-hsDNA (hsDNA-AO) reduces gradually at the maximum wavelength of 528 (531) nm in Figures 9 and 10. The experimental result indicates that the reaction competition between Cu-bipy and AO with hsDNA is conspicuous, and the bonding mode between Cu-bipy and hsDNA mainly includes insertion binding.

#### 3.4.4. Scatchard Method

**Figure 11:**
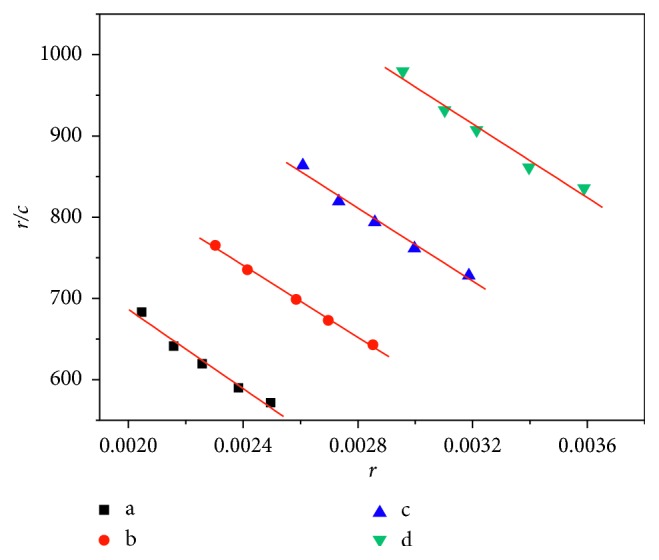
Scatchard plots of the interaction between Cu-bipy and hsDNA-AO (pH = 7.40, without NaCl). *c*(hsDNA) = 1.0 × 10^−5^ mol·L^−1^; Rt = *c*(Cu-bipy)/*c*(hsDNA); Rt = a, 0.00; b, 0.40; c, 0.80; d, 1.20.

**Figure 12:**
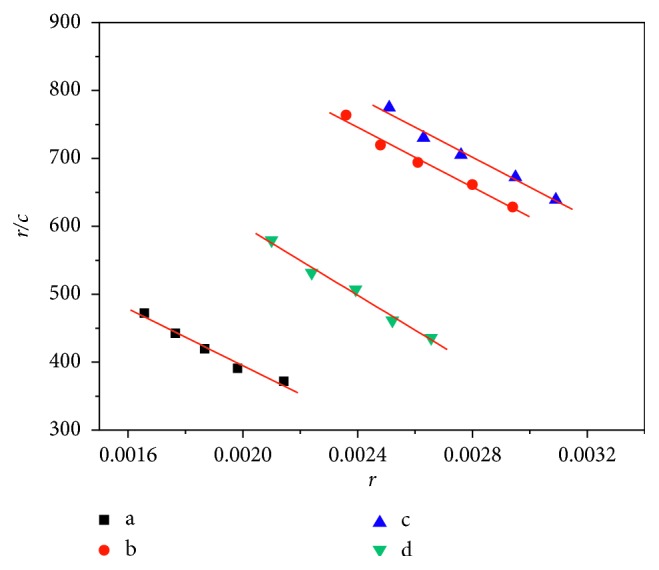
Scatchard plots of the interaction between Cu-bipy and hsDNA-AO (pH = 7.40, with NaCl). *c*(hsDNA) = 1.0 × 10^−5^ mol·L^−1^; Rt = *c*(Cu-bipy)/*c*(hsDNA); Rt = a, 0.00; b, 0.40; c, 0.80; d, 1.20.

The Scatchard equation (3) can be used to study the binding mode between hsDNA and AO with Cu-bipy, whose concentration is gradually changing.

(3) $$\begin{array}{c} \frac{r}{c}=K\left(n-r\right), \end{array}$$

where *r* is the mole number of AO bound per mole of DNA, *c* is the AO concentration, *K* is the binding constant, and *n* is the maximum value of a binding site with AO. Generally, if the *n* value in the absence of Cu-bipy is the same with the presence of Cu-bipy, the binding mode is an insertion mode. If the *K* value in the absence of Cu-bipy is the same with the presence of Cu-bipy, there is noninsertion in the binding mode. If the *K* value is different from the *n* value, the binding mode between Cu-bipy and hsDNA is a mixed mode of noninsertion and insertion binding. The Scatchard plots in the absence and the presence of sodium chloride are shown in Figures 11 and 12, and the data of *n* and *K* are listed in Table 5. It can be seen from Table 5 that both values of *n* and *K* vary with the concentrations of Cu-bipy. The results show the presence of the mixed interaction. The *n* values in the presence of sodium chloride are lower than that of no sodium chloride, and this indicates that there is an electrostatic interaction between Cu-bipy and hsDNA.

#### 3.4.5. Influence of Phosphate Group

**Figure 13:**
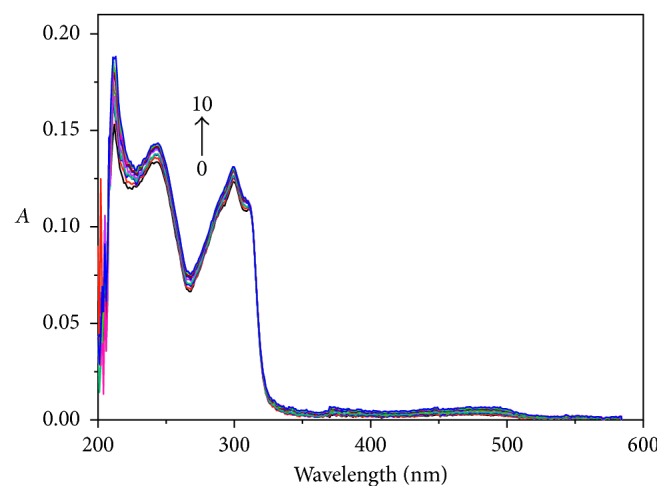
Influence of phosphate on the UV-Vis spectra of Cu-bipy (pH 7.40). *c*(Cu-bipy) = 1.0 × 10^−5^ mol·L^−1^; *c*(Na_2_HPO_4_) = 0.00, 0.33, 0.67, 1.00, 1.33, 1.67, 2.00, 2.33, 2.67, 3.00, 3.33, 3.67, 4.00, 4.33, 4.67, and 5.00 × 10^−5^ mol·L^−1^, respectively.

The above conclusion is further demonstrated by the phosphate experiment. If Cu-bipy binds to phosphate radical, then there is an electrostatic interaction between Cu-bipy and hsDNA by changing the Na_2_HPO_4_ concentration while keeping the Cu-bipy concentration fixed. As shown in Figure 13, when the amounts of Na_2_HPO_4_ are increased, UV-Vis spectra of Cu-bipy are slightly changed. The result hints that the electrostatic interaction exists between Cu-bipy and hsDNA.

#### 3.4.6. Viscosity Measurements

**Figure 14:**
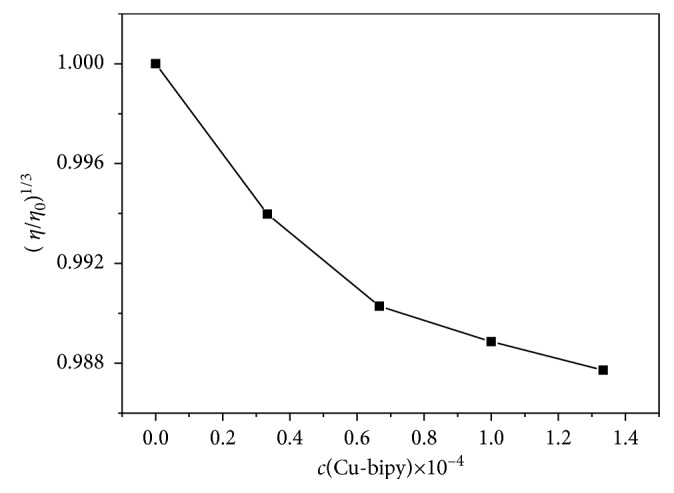
Effect of increasing amounts of Cu-bipy on the relative viscosity of hsDNA, *c*(hsDNA) = 1.00 × 10^−4^ mol·L^−1^.

The viscosity measurements of complexes at different concentrations can obtain useful data for identifying binding mode [41, 52]. If a micromolecule is inserted in the interspace of base pairs, the DNA helix will be extended because the separated base pairs can accommodate the bound ligand. Conversely, the viscosity will not increase if the binding with DNA is in other ways; the groove binding does not obviously change viscosity, whereas a partial intercalation of the complex causes a bend in the DNA helix, reducing its viscosity [53, 54]. The viscosity was determined by the fixed hsDNA concentration and changing the Cu-bipy concentration in the experiment. In Figure 14, the relative viscosity of hsDNA reveals a consistent decrease during the addition of Cu-bipy, which may be due to partial inserting of the complex. According to the result of viscosity measurement, the interaction between Cu-bipy and hsDNA is in insertion mode.

## 4. Conclusions

The complex [Cu(bipy)(H_2_O)_2_(SO_4_)]_*n*_ was synthesized by hydrothermal method and characterized by EA, single crystal X-ray diffraction, FTIR, and TG-DSC. The complex crystallizes in the monoclinic system with *C*2/*c* space group. The Cu(II) was hexacoordinated by two N atoms and four O atoms, forming a slightly distorted octahedron, and bridged by sulfato groups into polymeric chains. Under the physiological pH, the interaction between the complex and hsDNA was studied with AO as a fluorescent probe by spectral method. The interaction mechanism of the complex with hsDNA is electrostatic and intercalative binding. The calculated thermodynamic parameters indicate that the interaction of the complex and hsDNA is driven by entropy. The influence of phosphate radical and Scatchard method reveals that the complex is combined with hsDNA in the electrostatic and intromittent modes.

## Figures and Tables

**Table 1:** Crystal data and structure refinement parameters for the title complex.

Empirical formula	CuC_10_H_12_O_6_N_2_S
Formula weight (g·mol^−1^)	351.82
Temperature (K)	296(2)
Wavelength (Å)	0.71073
Crystal system	Monoclinic
Space group	*C*2*/c*
*a* (Å)	15.1279(7)
*b* (Å)	12.4488(6)
*c* (Å)	6.9987(3)
*β* (°)	105.9576(13)
*V* (Å^3^)	1267.23(10)
*Z*	4
Calculated density (g·cm^−3^)	1.844
Absorption coefficient (mm^−1^)	1.916
*F*(000)	716
Crystal size (mm^3^)	0.374 × 0.199 × 0.117
*θ* range for data collection (°)	3.273–27.639
Index ranges	−19 ≤ *h* ≤ 19, −16 ≤ *k* ≤ 16, −9 ≤ *l* ≤ 8
Reflections collected/unique	9535
Data/restraints/parameters	1473/3/97
Goodness of fit on *F*^2^	0.974
Final *R* indices (*I* > 2*σ* (*I*))	*R*_1_ = 0.0226, *wR*_2_ = 0.0579
*R* indices (all data)	*R*_1_ = 0.0238, *wR*_2_ = 0.0589
Largest differential peak and hole (e·Å^−3^)	0.356 and −0.442

**Table 2:** Selected bond lengths (Å) and angles (°) for the title complex.

Cu(1)–O(1)	1.9728(11)
Cu(1)–O(1)^#1^	1.9728(11)
Cu(1)–N(1)	1.9947(12)
Cu(1)–N(1)^#1^	1.9948(13)
Cu(1)–O(2)	2.455
Cu(1)–O(2)^#1^	2.455
O(1)^#1^–Cu(1)–O(1)	93.07(7)
O(1)^#1^–Cu(1)–N(1)	93.11(5)
O(1)–Cu(1)–N(1)	172.31(5)
O(1)^#1^–Cu(1)–N(1)^#1^	172.31(5)
O(1)–Cu(1)–N(1)^#1^	93.11(5)
N(1)–Cu(1)–N(1)^#1^	81.12(7)
O(2)–Cu(1)–O(1)	85.52
O(2)–Cu(1)–O(1)^#1^	92.25
O(2)–Cu(1)–N(1)	89.66
O(2)–Cu(1)–N(1)^#1^	92.79
O(2)–Cu(1)–O(2)^#1^	176.77
O(2)^#1^–Cu(1)–O(1)	92.25
O(2)^#1^–Cu(1)–N(1)	92.97
O(2)^#1^–Cu(1)–N(1)^#1^	89.66
O(2)^#1^–Cu(1)–O(1)^#1^	85.52

Symmetry transformations used to generate equivalent atoms: ^#1^−*x* + 2, *y*, −*z* + 1/2; ^#2^−*x* + 2, *y*, −*z* + 3/2.

**Table 3:** Hydrogen bond lengths (Å) and bond angles (°) for the title complex.

D–H⋯A	*d*(D–H)	*d*(H⋯A)	*d*(D⋯A)	∠DHA
O(1)–H(1W)⋯O(3)^#3^	0.852(9)	1.843(11)	2.6763(16)	165.4(19)
O(1)–H(1W)⋯S(1)^#3^	0.852(9)	2.926(16)	3.6599(12)	145.4(18)
C(5)–H(5)⋯O(1)^#1^	0.93	2.50	3.026(2)	115.9
C(2)–H(2)⋯O(2)^#4^	0.93	2.43	3.282(2)	152.1
O(1)–H(1)⋯O(3)^#1^	0.82	1.81	2.6198(16)	169.5
O(1)–H(1)⋯S(1)^#5^	0.82	2.74	3.4143(11)	140.8

Symmetry transformations used to generate equivalent atoms: ^#1^−*x* + 2, *y*, −*z* + 1/2; ^#2^−*x* + 2, *y*, −*z* + 3/2; ^#3^−*x* + 2, −*y* + 1, −*z* + 1; ^#4^−*x* + 2, −*y* + 2, −*z* + 1; ^#5^*x*, *y*, *z*−1.

**Table 4:** Thermodynamic parameters at two different temperatures.

*T* (K)	*K* ^⊝^ (L·mol^−1^)	Δ_r_*G*_m_^⊝^ (J·mol^−1^)	Δ_r_*S*_m_^⊝^ (J·mol^−1^·K^−1^)	Δ_r_*H*_m_^⊝^ (J·mol^−1^)
295.15	1.97 × 10^5^	−3.53 × 10^4^	115.26	−1280
313.15	1.55 × 10^4^	−2.96 × 10^4^	90.44	−1280

**Table 5:** Data from the Scatchard equation of the interaction between Cu-bipy and hsDNA.

Curve	*c*(Cu-bipy)/*c*(hsDNA)	NaCl (mol·L^−1^)	Scatchard	*K* (L·mol^−1^)	*n*
a	0.00	0	1173.6–2.44 × 10^5^*r*	2.44 × 10^5^	4.81 × 10^−3^
		0.50	813.4–2.09 × 10^5^*r*	2.09 × 10^5^	3.89 × 10^−3^
b	0.40	0	1176.5–1.81 × 10^5^*r*	1.81 × 10^5^	6.50 × 10^−3^
		0.50	1241.5–2.20 × 10^5^*r*	2.20 × 10^5^	5.64 × 10^−3^
c	0.80	0	1441.2–2.25 × 10^5^*r*	2.25 × 10^5^	6.41 × 10^−3^
		0.50	1318.6–2.21 × 10^5^*r*	2.21 × 10^5^	5.97 × 10^−3^
d	1.20	0	1641.4–2.27 × 10^5^*r*	2.27 × 10^5^	7.23 × 10^−3^
		0.50	1113.9–2.56 × 10^5^*r*	2.56 × 10^5^	4.35 × 10^−3^

## Data Availability

Crystallographic data have been deposited with the Cambridge Crystallographic Data Centre, CCDC, 12 Union Road, Cambridge CB2 1EZ, UK. Copies of the data can be obtained free of charge on quoting the depository CCDC-1028718 for the title complex (Fax: +44-1223-336-033; E-mail: deposit@ccdc.cam.ac.uk, http://www.ccdc.cam.ac.uk).
